# Short-Term Choriocapillaris Changes in Patients with Central Serous Chorioretinopathy after Half-Dose Photodynamic Therapy

**DOI:** 10.3390/ijms18112468

**Published:** 2017-11-20

**Authors:** Marco Nassisi, Carlo Lavia, Camilla Alovisi, Luca Musso, Chiara M. Eandi

**Affiliations:** Department of Surgical Science, University of Torino, 10126 Torino, Italy; m.nassisi@gmail.com (M.N.); carlo.lavia@googlemail.com (C.L.); camillaalovisi@gmail.com (C.A.); luk.musso@libero.it (L.M.)

**Keywords:** choriocapillaris, retina, choroid, central serous chorioretinopathy, photodynamic therapy

## Abstract

Background: Although photodynamic therapy (PDT) has become the standard treatment for central serous chorioretinopathy (CSC), its mechanism of action remains unclear. It is assumed that PDT induces short-term choriocapillaris (CC) occlusion and long-term choroidal vascular remodeling. In this paper, we describe the short-term CC changes induced by Half-Dose PDT (HD-PDT) in chronic CSC using optical coherence tomography-angiography (OCTA). Methods: This is a prospective interventional case series. Chronic CSC eyes underwent Spectral-Domain OCT, Fundus Autofluorescence, FA, ICGA (Heidelberg Spectralis, Heidelberg, Germany) and OCTA (RTVue XR Avanti with AngioVue; Optovue Inc., Fremont, CA, USA) before HD-PDT, with follow-up after one hour, one week, and one month. Vascular changes after PDT were analyzed within the CC layer. The CC vessel density was defined as the percentage of an area occupied by flow pixels, using Image J software to obtain measurements by applying a grey level threshold. All pixels with a grey level above the threshold were considered as indicators of blood flow. Results: 20 eyes of 19 patients were included. At baseline the mean CC vessel density was 94.87 ± 2.32%. It significantly differed from the density at 1 week and 1 month (92.79 ± 3.16% and 95.55 ± 2.05%, *p* < 0.001, respectively), but not with values at 1 h (94.8 ± 2.28%, *p* = 0.516). Conclusions: CC vessel density was significantly reduced at 1 week as compared with baseline, suggesting a possible short-term effect of PDT on CC perfusion. After 1 month however, the CC vessel density was even higher than the baseline, probably due to a CC recovery. OCTA seems to be useful in the visualization of CC vessels and in confirming the mechanism of action of PDT treatment in eyes with chronic CSC.

## 1. Introduction

Central serous chorioretinopathy (CSC) is a posterior segment disease that represents a common cause of vision threat in the middle-aged population. It is characterized by the presence of a localized and limited accumulation of fluid under the neurosensory retina through a defect of the retinal pigment epithelium (RPE) [1,2]. CSC often resolves spontaneously but sometimes recurs or becomes chronic [1,3].

The distinction between acute and chronic forms relies on the presence of persistent serous retinal detachment, extended RPE and photoreceptor atrophic changes correlated with a progressive visual loss over a period of 3–6 months [4,5,6]. This arbitrary demarcation has therapeutic implications as it usually determines the timing for intervention.

Classically, treatment is justified in case of persistent macular neuroretinal detachment or posterior cystoid retinal degeneration, history of multiple recurrences, history of CSC in the fellow eye with poor visual outcome, reduced visual acuity and rapid recovery required [7].

Up to now, there is no recognized standard therapy for chronic CSC which would allow for the prevention of progressive pigment epithelium and photoreceptor damage leading to irreversible visual impairment [8]. Although there are no phase 3 randomized clinical trials, photodynamic therapy (PDT) is now considered the treatment of choice for chronic CSC.

The efficacy of PDT—in particular half-dose or half-fluence PDT in eyes affected by CSC—has been widely studied and documented [9,10,11]. However, the mechanisms of the actions of PDT in chronic CSC remain unclear. Chan et al., speculated that PDT induces short-term choriocapillaris (CC) occlusion and long-term choroidal vascular remodeling [12]. These changes result in caliber normalization of dilated, congested, choroidal vessels and subsequent reduction of vascular hyperpermeability and leakage. Imaging techniques and histological findings have demonstrated the effects of vascular occlusion at the CC level due to PDT, even a few days after the treatment [13].

Recently, new imaging technologies have been developed and allow for new insights in the visualization of normal and pathologic vascularization. In particular, optical coherence tomography angiography (OCTA) is now able to individually visualize the superficial and deep retinal plexi, analyze the outer retina together and the CC layer without the need for dye injection [14]. OCTA findings in chronic CSC have been previously reported and have demonstrated that OCTA can detect dilated CC vessels and even choroidal neovascularization (CNV), not visible with other imaging techniques [15,16].

The aim of our study is to describe the short-term CC changes induced by Half-Dose PDT (HD-PDT) in the treatment of chronic CSC using OCTA.

## 2. Results

Using the Shapiro-Wilk test, all data sets resulted normally distributed (*p* > 0.05) [17].

### 2.1. Baseline Characteristics

Twenty eyes of 19 patients received half-dose PDT. Demographic data are shown in Table 1.

### 2.2. Visual Acuity

At baseline the mean best corrected visual acuity (BCVA) was 0.34 ± 0.23 LogMAR (Snellen equivalent: range 20/125–20/20; median 20/40). At one week and 1 month, BCVA was 0.31 ± 0.20 LogMAR (Snellen equivalent: range 20/100–20/25; median 20/40) and 0.26 ± 0.19 LogMAR (Snellen equivalent: range 20/100–20/25; median 20/40) respectively. There was no significant difference between BCVA at baseline and 1 week (*p* = 0.29). BCVA at the 1 month follow up visit was significantly different from the baseline (*p* = 0.02) (Table 2).

### 2.3. PDT

Complete resolution of subretinal fluid was observed in 12 eyes (60%) after 1 week and in 16 eyes (80%) after 1 month. Two cases achieved clinical healing at 3 months after treatment, while the two remaining eyes presented persistence of subretinal fluid and needed a second PDT treatment.

### 2.4. OCT

At baseline the mean central foveal thickness (CFT) was 348.3 ± 144.42 µm. At one hour, one week and one month it was 332.7 ± 112.87; 284.4 ± 67.08 and 249.75 ± 42.66 µm respectively. The CFT at baseline was significantly different from CFT at 1 week (*p* = 0.01) and 1 month (*p* = 0.005) but not at 1 h (*p* = 0.107) (Table 2).

The mean choroidal thickness (CHT) was 395.8 ± 89.61 µm. At one hour, one week and one month it was 397.1 ± 93.98; 381.9 ± 93.88 and 364.55 ± 108.5 µm respectively. The CHT at baseline was significantly different from CHT at one month (*p* = 0.009) but not at 1 h (*p* = 0.878) and 1 week (*p* = 0.134) (Table 2).

The mean CHT of fellow eyes was 284 ± 157.8 µm. It was statistically thinner than the affected eyes at any visit (*p* < 0.001).

### 2.5. OCTA

#### 2.5.1. Repeatability Assessment

Two different operators (M.N. and C.L.) performed all CCVD measurements. No significant difference was found between the two datasets (*p* = 0.114) and the intraclass correlation coefficient (ICC) was 0.982 (95% confidence interval [CI]: 0.972–0.988).

Mean values between the two measurements were used for further analysis.

#### 2.5.2. Affected Eyes

At baseline the mean CC vessel density was 94.87 ± 2.32%. It significantly differed from the density at 1 week (92.79 ± 3.16%, *p* < 0.001) and at 1 month (95.55 ± 2.05%, *p* < 0.001), but not with values at 1 h (94.8 ± 2.28%, *p* = 0.516) (Table 2).

**Figure 1:**
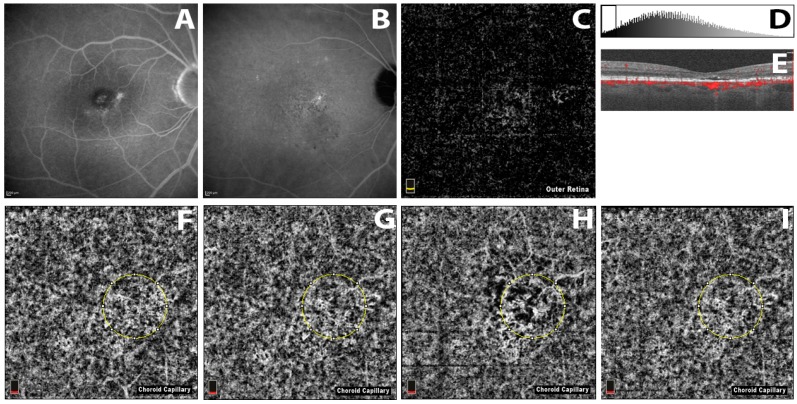
(**A**,**B**) Fluorescein Angiography (FA) and Indocyanine Green Angiography (ICGA) of an eye affected by chronic central serous choriorethinopathy (CSC); (**C**–**I**) Choriocapillaris (CC) vessel density evaluation before and after photodynamic (PDT) treatment in eyes affected by chronic CSC; (**C**) “Outer retina” Optical Coherence Tomography Angiography (OCTA) slab between 70 µm below inner plexiform layer and 30 µm below the RPE reference; (**F**–**I**) 30 µm slab OCTA CC image with the analyzed circle area (1 mm diameter) at baseline (**F**); 1 h (**G**); 1 week (**H**) and 1 month (**I**) after PDT; (**D**) Histogram analysis with the exclusion of all pixels having grey values below the individual noise level seen in (**C**); (**E**) OCTA B-scan of the baseline.

CC vessel density was significantly reduced at 1 week as compared with baseline, suggesting a possible short-term effect of PDT on CC perfusion. At 1 month, however, CC vessel density was even higher than baseline, probably due to a CC recovery (Figure 1).

No significant correlation was found between CFT and CCVD measurements (Spearman coefficient: −0.183, *p* = 0.103).

#### 2.5.3. Comparison with Fellow Eyes

For the analysis of fellow eyes, we excluded 3 patients because of bilateral CSC. We evaluated 16 healthy eyes from 16 patients. The mean CC vessel density for fellow eyes was 98.31 ± 0.98% and it differed significantly from data of the corresponding pathologic eyes at all stages of follow-up (*p* < 0.001).

This finding may be explained by a CC compression by bigger choroidal vessels occurring in affected eyes as compared with their healthy fellow eyes.

## 3. Discussion

Chronic CSC is a cause of progressive visual loss due to the persistence of the neuroepithelium serous detachment [18]. Even though there is no approved therapy for such a disease, PDT treatment with verteporfin showed good efficacy in resolving the subretinal fluid, even after long term follow-up [9,19].

In this study we demonstrated the short term PDT effects at the level of the choroid and the CC vessels in particular.

In the present series, significant CFT and CHT reduction together with BCVA improvement were observed starting one month after PDT, as expected. In our study the resolution of subretinal fluid at one month was observed in 16 eyes (80%). Clinical healing was achieved three months after the treatment in two cases, while the two remaining eyes showed persistent subretinal fluid requiring a second PDT treatment. Our results are similar to those of previous studies using half-dose PDT. In particular, Chan et al., showed a complete resolution of subretinal fluid in 79.5% at one month and 94.9% at 12 months, with only one recurrence [20]. More recently, Nicolò et al., reported similar results, with a success rate of 86.2% at one month and 100% at 12 months [11].

In this study we measured the CC vessel density in eyes with chronic CSC. We found a reduction of the CC vessel density one week after PDT treatment, while one month later it improved compared to the baseline. These observations support the hypothesis proposed to explain the mechanism of the actions of PDT treatment in CSC. In fact, PDT treatment seems to be able to induce some narrowing and remodeling of the choroidal vessels, thereby reducing choroidal exudation and, consequently, subretinal fluid [21].

OCTA is a new non-invasive imaging technique with interesting clinical applications and which is easy to perform. Recently, Xu et al. [22] have provided a qualitative short-term evaluation of the CC in patients with chronic CSC who underwent half-dose PDT. In their study CC changes after PDT support the hypothesis of short-term CC hypoperfusion after PDT, in line with our findings. Other authors described similar observations in a Japanese cohort [23].

In CSC-fellow eyes, the CC vessel density was similar to that found by Alten et al., in an age-matched group of normal eyes [24]. The CC vessel density found in treated eyes was somewhat inferior compared to fellow eyes: Even though the choroid is thicker, a CC compression from bigger choroidal vessels may have occurred in the affected eyes. Costanzo et al., studying the OCTA features in patients affected by CSC, reported a rarefaction of the CC plexus and the presence of “dark areas”, probably due to focal atrophy secondary to compression by the enlarged choroidal vessels [25,26].

The increase in “dark areas” observed one week after PDT may be related to the reabsorption of subretinal fluid, allowing a better visualization of the CC with higher contrast and less signal fragmentation. Nevertheless, the reduction of subretinal fluid (SRF) and CFT observed one week and one month after the treatment were similar, while CC vessel densities were rather different. We can therefore postulate that artifacts due to the possible SRF masking effect would have had similar effects at these timelines, suggesting that CC vessel density changes may have occurred between seven and 30 days after PDT. Furthermore, no correlation was found between CFT and CCVD. The increase of dark areas might even be related to the occlusion of the CC vessels, as an effect of PDT treatment. This theory has been proven by Schlötzer-Schrehardt et al., who demonstrated a selective damage on CC endothelial cells [12,27] with a preservation of bigger choroidal vessels. The damage induced by PDT may be responsible for the significant decrease in CC vessel density observed one week after the treatment. As the effect of PDT involves not only the targeted points of vascular focal loss, but even the surrounding areas, large dark areas are clearly visible in many OCTA scans.

It is possible that both focal atrophy due to compression by enlarged choroidal vessels and CC occlusion due to PDT may concur in producing these “dark areas” in CSC patients but further investigations are needed in order to clarify this point.

One month after the treatment, a significant increase in CC vessel density was observed, with values even higher than at baseline. This finding could be related to a process of recovery at the CC level after PDT. The effects of PDT on the choroid resulted in an increased expression of vascular endothelial growth factor (VEGF) and its receptors (VEGF-R) in the treated area, probably due to hypoxia and endothelial cells death [28]. Husain et al., demonstrated CC reperfusion in the normal choroid occurring within seven weeks from treatment, while Schlötzer-Schrehardt found CC recanalization as early as one week [29]. Moreover, CC vessel density recovery could be related to a decreased compression exerted by larger choroidal vessels.

The present study has several limitations and some considerations are dutiful.

Due to the present lack of standardization in CC analysis, vessel density results should be interpreted with caution and are not comparable to those reported in other studies using different thresholding and calculation methods [30,31].

3 × 3 mm scan dimension was preferred to the 6 × 6 mm because of its higher resolution, especially for CC evaluation and processing. Moreover, the evaluated area was small and was the same for every patient at all follow-up. Image artifacts were reported more often when using 3 × 3 mm scan, and these artifacts were also more frequent in elderly people with poor fixation [24].

In this study the standard 30 µm CC slab was used to evaluate CC vessel density with OCTA. Even if a manual segmentation was performed in cases of unsatisfying automated procedure, a different slab could have been chosen. The 30 µm slab at the CC level includes even parts of the larger choroidal vessels: A “tailored” slab could have brought to different and maybe more accurate results. However, Alten et al., studying reticular pseudodrusen (RPD) features using the OCTA, found no significant differences between 10 and 30 µm slabs within the CC in affected patients [24]. Due to different CC and choroid conditions in CSC and RPD, these considerations are only hypothesis and further research is needed.

Patients with pigment epithelium detachment (PED) or posterior cystoid retinal degeneration were excluded from the study. As the aim of the article was to evaluate the CC structural changes after HD-PDT in patients with chronic CSC, patients with PED were excluded due to the segmentation difficulties deriving from the CC displacement observed in PED, as previously reported [32]. Cystoid retinal degeneration in CSC is a recognized complication of chronic CSC and is associated with a severe reduction of visual acuity as a result of long-term disease [33]. In this condition it could have been impossible to obtain good quality images from those patients, hence the prior exclusion from the study.

## 4. Materials and Methods

This prospective interventional case series included 20 eyes of 19 consecutive patients (18 males, mean age 51.8 ± 8.01 years, range 33–62 years) affected by chronic CSC that underwent half-dose PDT with verteporfin at our Eye Clinic in Turin, Italy.

The study was approved by the Institutional Review Board of the University of Turin (CS/723, 14 March 2016, Comitato Etico Interaziendale A.O.U. Città della Salute e della Scienza di Torino—A.O. Ordine Mauriziano di Torino—A.S.L. TO 1). Informed consent for PDT and for clinical research was obtained from all patients and the study was conducted in accordance with the provisions stated in the Declaration of Helsinki.

Chronic CSC was defined as persistence of subretinal fluid involving the fovea for six months or more.

Inclusion criteria of the study were:

1. Snellen BCVA of 20/200 or better
2. Presence of active leakage on fluorescein angiography (FA) and choroidal vascular hyperpermeability on indocyanine green angiography (ICGA)
3. Absence of CNV evidence on OCTA, FA and ICGA
4. Absence of PED or posterior cystoid retinal degeneration
5. Absence of any previous treatment
6. Absence of any other chorioretinal disease
7. Absence of media opacities

BCVA was measured at baseline and after one month visit using the Early Treatment Diabetic Retinopathy Study (ETDRS) charts by certified examiners (M.N. and C.L.). Spectral Domain OCT (SD-OCT), Fundus Autofluorescence (FAF), FA, ICGA (Heidelberg Spectralis, Heidelberg, Germany) and OCTA (RTVue XR Avanti with AngioVue; Optovue Inc., Fremont, CA, USA) were performed by certified examiners after pupillary dilatation (C.M.E., C.A.).

Cross-line SD-OCT scan confirmed the presence of macular serous detachment. CFT was measured using the Retina Map pattern and the provided ETDRS grid in the central millimeter. Each section was obtained using eye tracking ART (Automatic Real-Time) and 100 scans were averaged to improve the signal-to-noise ratio. Enhanced depth imaging (EDI) technique was used to calculate CHT using the attached measuring software. Central choroidal thickness was measured under the center of the fovea as previously described [34]. The subfoveal CHT was measured from the outer surface of the line formed by the RPE to the inner surface of the observed sclera and calculated as the mean value between vertical and horizontal line scans.

The baseline visit was performed within one week before the PDT treatment. All patients underwent a complete ophthalmic examination including: BCVA, anterior segment examination, intraocular pressure measurement with Goldmann applanation tonometry and dilated fundus biomicroscopy. FAF, FA and ICGA were also performed. SD-OCT and OCTA examination were performed right before the PDT treatment, one hour after, and after one week and one month visit.

Baseline examinations were performed even in fellow eyes. If no signs of CSC or any other ocular pathology was detected in fellow eyes, they were used to make comparisons in terms of CHT and CC vessel density.

Patients satisfying the inclusion criteria, underwent ICGA guided HD-PDT (3 mg/m^2^) as previously described [35]. Briefly, verteporfin (Visudyne; Novartis AG, Basel, Switzerland) was infused for 10 min followed by 83 s of laser treatment at 693 nm (total energy 50 J/cm^2^) delivered 15 min after the beginning of the infusion. HD-PDT was targeted to the areas of choroidal hyper-permeability visible on ICGA supposed to cause the serous detachment.

### 4.1. OCT-Angiography

Optovue RTVue XR Avanti Spectral-Domain (SD) OCT with AngioVue was used to obtain amplitude-decorrelated angiography images. The instrument has an A-scan rate of 70,000 scans per second, a light source centered at 840 nm and a bandwidth of 45 nm. Each OCTA volume contains 304 × 304 A-scans and is acquired over three s. Two consecutive orthogonal OCTA volumes are acquired to perform a motion correction which automatically minimize the motion artifacts due to microsaccades (i.e., motion correction technology [MCT]). Using the Split-spectrum method (SSADA), measurement noise was reduced by splitting the signal into eleven wavelengths.

An algorithm of the software (Version 2015.100.0.35; Optovue Inc., Fremont, CA, USA) analyses and calculates each pixel location and combines horizontal and vertical scans averaging the decorrelation values to create a 3-dimensional angiographic data cube. The modifications in reflectivity are directly linked to blood flow. The software automatically segments the tissue in four layers: The superficial layer (from the inner limiting membrane to the inner nuclear layer), the deep layer (from the inner nuclear layer to the outer plexiform layer), the outer retina layer (from the outer plexiform layer to the retinal pigment epithelium) and the CC layer.

Vascular changes after PDT treatment were analyzed within the CC layer. The visualization of the CC layer was obtained using the standard setting of the instrument which gives a 30 µm-slab between 30 and 60 µm below the inner RPE reference. As slabs were automatically-generated, all segmentations were checked and eventually manually adjusted for accuracy.

3 × 3 mm OCTA acquisitions were centered in the leakage area on FA or hyperpermeable spot on ICGA images before PDT treatment; the follow-up mode was then used for the following examinations. Images showing an inadequate signal (signal strength index < 60) or evidence of signal blockage on the En-Face scans of the CC were excluded.

Using the software Image J (National Institutes of Health; http://imagej.nih.gov/ij/) a 1-mm-diameter circle was traced in the center of the 3 × 3 mm OCT angiograms. The appropriate position of the circle was verified using the angiographic images and the vascular plexi of the inner retina imaged with OCTA as references.

**Figure 2:**
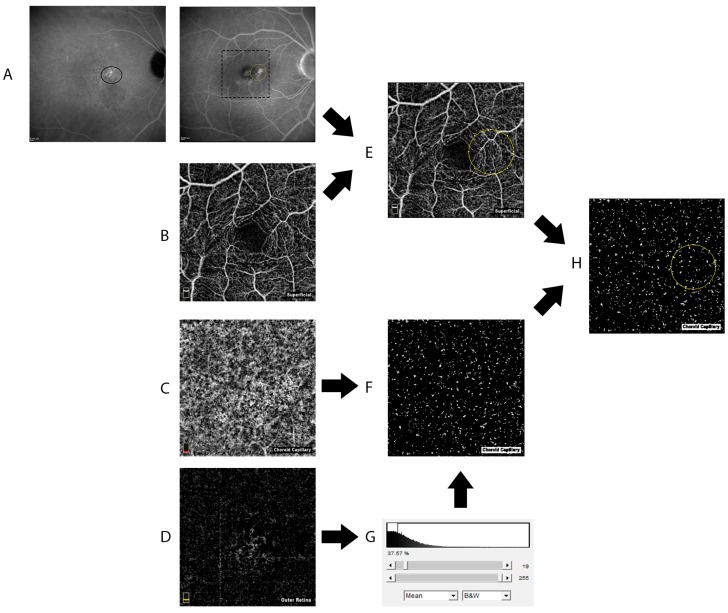
(**A**) The Indocyanine Green Angiography (ICGA, left) of an eye affected by chronic central serous choriorethinopathy (CSC) was used as reference to guide the photodynamic (PDT) treatment (black ellipsoid line); (**E**) The appropriate position of the circle on the optical coherence tomography angiography (OCTA) image was verified comparing the Fluorescein Angiography (FA; (**A**) right) image and the vascular plexus of the inner retina imaged with (OCTA) (**B**); (**D**) “Outer retina” OCTA slab between 70 µm below inner plexiform layer and 30 µm below the retinal pigment epithelium (RPE) reference was used to define the noise as the mean grey level of the picture (**G**). The mean grey level was used as a threshold to apply to the 30 µm slab OCTA choriocapillaris (CC) image (**C**), obtaining a binarized image where all pixels above the threshold (in black) were considered as indicators of blood flow (**F**). Using the previous superficial slab (**E**) as reference, the circle was placed on the binarized image in the same position (**H**). The area of the black pixels was automatically measured by ImageJ software.

Mean gray levels of the four layers of each image were assessed using Image J software. As previously described [24], the CC vessel density was defined as the percentage area occupied by flow pixels obtained by applying a grey level threshold. The grey level threshold was used to reduce the noise and was defined as the average signal of the outer retinal layer, where normally no vessels are found. All pixels with a grey level above the threshold were considered as indicators of blood flow (Figure 2). In order to enhance reliability of our data we performed two consecutive OCTA measurements in each eye by two different operators (MN and CL). Mean values were used for further analysis. CC vessel density is expressed as percentage.

### 4.2. Data Analysis and Statistic

All data are presented as Mean ± Standard Deviation.
The Shapiro-Wilk test was used to verify the normal distribution of data [17].
Differences between data sets were evaluated with paired *t*-test.
Repeatability between the two measurements from different operators was assessed evaluating the ICC.
The Spearman coefficient was calculated to assess the correlation between CCVD and CFT.
*p* value < 0.05 was considered significant.

## 5. Conclusions

In conclusion, the short-term analysis of the CC after PDT showed a decrease in vessel density one week after the treatment, probably due to capillary occlusion subsequent to treatment. The vessel density increase observed at one month may be due to CC recovery related to VEGF and VEGF-R overexpression at the treatment site. OCTA seems to be useful in the visualization of the CC vessels and in confirming the mechanism of action of PDT treatment in eyes with chronic CSC.

## Figures and Tables

**Table 1:** Baseline demographic characteristics.

Characteristic	Value ± SD
Mean Age ± Standard Deviation (Years)	51.8 ± 8.01
Sex (Male/Female)	18/1
Mean duration of CSC ± Standard Deviation (Years)	3.34 ± 1.40
Patients with bilateral CSC (*n*)	3

**Table 2:** Mean Best Corrected Visual Acuity (BCVA), Central Foveal Thickness (CFT), Choroidal Thickness (CHT) and Choriocapillary Vessel Density (CCVD) in 20 eyes with Chronic Central Serous Chorioretinopathy treated with half-dose photodynamic therapy. SD: standard deviation; 1 h: 1 hour; 1 w: 1 week; 1 m: 1 month.

Characteristic	Baseline	1 h	*p* (Baseline vs. 1 h)	1 w	*p* (Baseline vs. 1 w)	1 m	*p* (Baseline vs. 1 m)
BCVA ± SD (LogMAR)	0.34 ± 0.23	*N*/*A*	*N*/*A*	0.31 ± 0.20	0.287	0.26 ± 0.19	0.016
CFT ± SD (μm)	348.3 ± 144.42	332.7 ± 112.87	0.107	284.4 ± 67.08	0.01	249.75 ± 42.66	0.005
CHT ± SD (μm)	395.8 ± 89.61	397.1 ± 93.98	0.878	381.9 ± 93.88	0.134	364.55 ± 108.5	0.009
CCVD ± SD (%)	94.87 ± 2.32	94.8 ± 2.28	0.516	92.79 ± 3.16	<0.001	95.55 ± 2.05	<0.001
